# Community Involvement in an Outbreak—One Year on for Mpox

**DOI:** 10.1093/cid/ciad745

**Published:** 2023-12-07

**Authors:** Ashleigh Cheyne, Ian Muchamore, Harun Tulunay, Amanda Rojek, Leon Peto, Peter Horby

**Affiliations:** Nuffield Department of Medicine, Pandemic Sciences Institute, University of Oxford, Oxford, United Kingdom; PLATINUM Community Advisory Panel, Nuffield Department of Medicine, Oxford, United Kingdom; PLATINUM Community Advisory Panel, Nuffield Department of Medicine, Oxford, United Kingdom; Nuffield Department of Medicine, Pandemic Sciences Institute, University of Oxford, Oxford, United Kingdom; Nuffield Department of Population Health, University of Oxford, Oxford, United Kingdom; Nuffield Department of Medicine, Pandemic Sciences Institute, University of Oxford, Oxford, United Kingdom

To the  Editor—We read with interest the article by Hazra and Cherabie [1], who conclude that the classification of mpox as a sexually transmitted infection would only worsen stigma faced by those affected. The authors provide examples of US initiatives to reduce stigma and emphasize educating communities on transmission dynamics and prevention methods. There is no mention of what affected communities think of mpox as an sexually transmitted infection, despite previous publications on this question [2], and no details on how to effectively involve communities in such discussions. Mpox-affected communities should be at the heart of this discourse. We call for a change in the global mpox response to be more community led, through the example of involving the community in a UK clinical treatment trial as a step toward this.

The UK mpox outbreak began in May 2022, with >3700 cases recorded, and has disproportionately affected gay, bisexual, and other men who have sex with men, who have been stigmatized as a result [3]. Between 38%–50% of those affected by mpox have also been with human immunodeficiency virus (HIV) [4]. The PLATINUM trial is a national UK trial to evaluate the safety and efficacy of tecovirimat in nonhospitalized patients with mpox. Early and rapid community leadership led to community involvement in the PLATINUM trial (Table 1).

**Table 1.** Community Involvement Activities in the PLATINUM Trial

*Activity*	*Activity Description*	*Impact on Trial*
*CAP*	Includes persons recovered from mpox, sexual health workers who have treated mpox patients, gay and bisexual, and other men who have sex with men, and people with HIV; contributed to study protocol, participant information sheet, consent form, patient recruitment poster and flyer, participant questionnaire, press release, and website	Increased inclusivity and appropriateness of language used in patient-facing materials
*Raising awareness*	Presentation of trial at conferences by community members (eg, HIV Glasgow); interview with community podcast “What the Pox?”; hosting a stall at Manchester Pride festival 2023 to provide information about the trial to festival attendees	Identification of potential avenues for raising trial awareness that are outside routine trial recruitment strategies; increased community awareness about the trial
*Training*	Training provided to CAP members on trial background, planned future training on scientific publications	Increased community member understanding of the trial and research in general
	Community-delivered training in stigma and HIV history to trial team as a result of steering committee recommendation	Whole trial team invited to training; attendees gained greater understanding of the stigma faced by groups at risk of mpox and appropriate language and terminology to use when engaging with those communities
*Community representation in steering committee*	A CAP member is a member of the trial steering committee; training in stigma and HIV history was recommended by the community representative	Community voice present in trial governance
*Communication with clinics*	Advice from CAP on how to recruit potential participants in clinics across the country	Patient-centered and language-appropriate recruitment posters and flyers
*Community organization engagement*	Support from Terrence Higgins Trust, UK Community Advisory Board, Positively UK, NAM AIDSMAP, Prepster, and HIV i-Base; HIV i-Base, Positively UK, and the British HIV Association have included the trial on their websites	Increased community and healthcare worker awareness of the trial, including diverse high-risk community groups
*Trial launch*	CAP reviewed the press release, provided a statement within the release, and tweeted about the trial launch alongside supporting organizations; this, together with trial team and university tweets, potentially reached >1.4 million people, and the trial was covered by community media channel PinkNews	Improving awareness for potential participants beyond those normally reached by research organization engagement

Abbreviations: CAP, Community Advisory Panel; HIV, human immunodeficiency virus.

Early community involvement was enabled by trial leadership and funders prioritizing community engagement. Support was provided by community organizations and a rapidly established community advisory panel, including individuals previously treated for mpox and those with HIV. Examples of their impact included changing the trial protocol to include emergency contact numbers for participants and enhancing recruitment and communication. Despite these early initiatives, challenges remained. The extensive time for study approvals meant that the peak mpox case numbers had passed before the study started.

There have also been mounting feelings of discontent, reflecting the frustration in the community of gay, bisexual, and other men who have sex with men toward the national UK mpox response. In contrast to the United States, no single government representative for the mpox response was elected to tackle the outbreak, resulting in lack of coordination across intervention efforts. Instead, and consistent with the US response as noted by Hazra and Cherabie [1], sexual health clinics that were already stretched and underfunded have had to manage the outbreak response, such as vaccinations and referrals to trials like PLATINUM.

For years there have been calls for greater involvement of communities in our public health responses, with accessible guidance on how to do this in fields such as in HIV research [5], and yet coordinated community-centered responses are still lacking. The activities highlighted in the PLATINUM trial are a step toward correcting this. However, significant improvements need to be made to further support community engagement in response to outbreaks like mpox, such as standardizing the involvement of communities in studies and discussions such as the one outlined by Hazra and Cherabie [1], and nominating leaders of contact for outbreak responses. Greater meaningful, and continuous community engagement will ensure that future trials are as effective as possible and improvements in care and control are achieved.

## Notes

***Author contributions.*** A. C., A. R., L. P., and P. H. wrote the manuscript, and all authors read and approved it.

***Acknowledgments.*** A. C., I. M., and H. T. wrote the article on behalf of the PLATINUM Community Advisory Panel (which includes I. M., H. T., and other anonymous members), A. R., L. P., and P. H. contributed to the engagement activities outlined in this article. The authors thank the community organizations that supported these activities: Terrence Higgins Trust, UK Community Advisory Board, Positively UK, NAM AIDSMAP, Prepster, and HIV i-Base.

***Disclaimer.*** The views expressed are those of the authors and not necessarily those of the National Institute for Health and Care Research or the Department of Health and Social Care.

***Financial support*.** This work was supported by the National Institute for Health and Care Research (NIHR) (grant NIHR135639 to P. H.), and SIGA Technologies (provision of tecovirimat and matching placebo for the PLATINUM trial).
